# Coverage of actionable alterations in non-small cell lung cancer by hybrid capture-based FoundationOne^®^CDx compared with amplicon-based OncomineDX

**DOI:** 10.1093/oncolo/oyaf390

**Published:** 2025-11-20

**Authors:** Kuei-Ting Chen, Vidyalakshmi Sethunath, Ericka Ebot, Brennan Decker, Amaya Gasco, Jeffrey S Ross, Andreas M Heilmann, Serenedy Smith, Katherine Lofgren, Richard S P Huang

**Affiliations:** Foundation Medicine, Inc., Boston, MA 02210, USA; Foundation Medicine, Inc., Boston, MA 02210, USA; Foundation Medicine, Inc., Boston, MA 02210, USA; Foundation Medicine, Inc., Boston, MA 02210, USA; Foundation Medicine, Inc., Boston, MA 02210, USA; Foundation Medicine, Inc., Boston, MA 02210, USA; Upstate Medical University, Syracuse, NY, USA; Foundation Medicine, Inc., Boston, MA 02210, USA; Foundation Medicine, Inc., Boston, MA 02210, USA; Foundation Medicine, Inc., Boston, MA 02210, USA; Foundation Medicine, Inc., Boston, MA 02210, USA

**Keywords:** actionable alterations, non-small cell lung cancer, hybrid capture, amplicon-based

## Abstract

**Introduction:**

FoundationOne^®^CDx (F1CDx) and Oncomine Dx target test (ODxTT) are commercially available oncology biomarker assays that apply different next-generation sequencing methodologies used to identify predictive markers for therapy selection in non-small cell lung cancer (NSCLC). We hypothesized that F1CDx can detect a higher number of actionable alterations compared with the ODxTT assay.

**Materials and Methods:**

We compared the prevalence of genomic alterations (GA) in a real-world NSCLC cohort tested by F1CDx to the theoretical detection by ODxTT, based on publicly available coverage specifications. The clinical actionability of GA was assessed based on NCCN treatment guidelines and FDA oncology drug approvals.

**Results:**

*KRAS* G12C, *EGFR* mutations, and *MET* mutations were among the most frequent actionable alterations detected in a large genomic dataset of NSCLC data assayed by the F1CDx test. The ODxTT specifications indicate that ODxTT detects all *BRAF* and *KR*AS FDA-approved alterations detected by F1CDx and subsets of the following clinically significant markers: *EGFR* 83%, *RET* 76%, *ROS1* 74%, and *ERBB2* 38%. In addition, ODxTT had no coverage for actionable genomic findings in *ALK*, *MET*, *NTRK1/2/3*, TMB, or MSI-High status. In this study, the ODxTT assay may fail to report at least one of the clinically actionable biomarkers in 42% of tissue samples compared to F1CDx.

**Discussion:**

In summary, in advanced NSCLC, hybrid capture-based NGS, such as F1CDx, may capture more actionable genomic alterations as compared with ODxTT, an amplicon-based NGS.

Implications for Practice Hybrid capture-based comprehensive genomic profiling, such as FoundationOne^®^CDx (F1CDx), identifies a broader spectrum of actionable biomarkers in advanced non-small cell lung cancer than amplicon-based assays like Oncomine Dx Target Test (ODxTT). In this study, ODxTT may fail to report at least one clinically actionable biomarker in 42% of tissue samples compared to F1CDx, highlighting that reliance on smaller amplicon-based panels can miss alterations directly impacting therapy selection. Incorporating hybrid capture-based NGS into routine diagnostics may improve identification of patients eligible for targeted and immunotherapies, supporting more personalized NSCLC treatment.

## Introduction

Next-generation sequencing (NGS)-based comprehensive genomic profiling (CGP) has become the standard-of-care diagnostic approach for treatment selection in advanced non-small cell lung cancer (NSCLC).^1^^,^^2^ There are numerous oncogenic driver genomic alterations in advanced NSCLC that have established clinical utility to guide treatment decisions, including *EGFR* mutations, *ERBB2* activating alterations, *ALK* rearrangements, *ROS1* rearrangements, *BRAF* mutations, *KRAS* mutations, *RET* rearrangements, *MET* exon 14 skipping mutations, *NTRK1/2/3* gene fusions.^3-5^ In comparison to single-gene testing, NGS enables multiplexed detection of genomic alterations across numerous genes from a single sample and assay, which reduces cost and turnaround times.^6^^,^^7^ In fact, NGS is recommended as the preferred molecular testing methodology by the National Comprehensive Cancer Network (NCCN) Guidelines in NSCLC.^4^

NGS-based profiling involves enrichment of specific genomic regions of interest from the background of the entire genome.^7^ This targeted sequence enrichment is generally achieved by using two major approaches: hybrid capture-based or polymerase chain reaction (PCR) amplicon-based techniques.^7^^,^^8^ These approaches have been extensively reviewed. Amplicon-based NGS is limited by the constraints of primer multiplexing, which restricts the simultaneous targeting of multiple genes. In contrast, hybrid capture-based NGS is not subject to these limitations.^7^^,^^9^ Hybrid capture-based NGS allows for a broad range of gene sequencing providing extensive genetic information, including exonic and intronic mutations, gene rearrangements, homozygous loss and amplifications.^10^ Amplicon-based NGS offers sequencing of a more narrow range of genes, focusing on point mutations in hotspot regions of the genome.^10^

There are currently two NGS-based diagnostics that are FDA-approved as companion diagnostics for ≥5 FDA-approved targeted therapies in NSCLC: hybrid capture-based or amplicon-based NGS techniques.^11^^,^^12^ The first of these FDA-approved NGS test, FoundationOne^®^CDx (F1CDx^®^), is a hybrid capture-based NGS test approved as a companion diagnostic of 14 unique targeted therapies, inclusive of combination therapies, specifically in NSCLC.^13^ Additionally, FoundationOne^®^CDx (F1CDx^®^) is also approved as a companion diagnostic for three other unique therapies in all solid tumors including NSCLC.^13^ Oncomine Dx (ODxTT) is an amplicon-based NGS test approved as a companion diagnostic for 7 targeted therapies utilized for treatment of advanced NSCLC.^12^ Herein, we sought to compare a hybrid capture-based NGS assay (F1CDx) with an amplicon-based NGS ODxTT (with *in silico* methods) in identifying clinically actionable biomarkers in NSCLC.

## Methods

### Comprehensive genomic profiling

F1CDx is a DNA based comprehensive genomic profiling (CGP) assay that utilizes a hybrid capture-based NGS approach targeting 324 genes and was performed in a Clinical Laboratory Improvement Amendments (CLIA)-certified, CAP (College of American Pathologists)-accredited laboratory (Foundation Medicine Inc.) utilizing FFPE tissue samples, as previously described.^14^ From 2018 January to 2022 June, 55 999 samples from research consented patients with NSCLC were received at Foundation Medicine for routine clinical care. Only samples that passed quality control checks were included in the analysis. Approval for this study, including a waiver of informed consent and Health Insurance Portability and Accountability Act waiver of authorization, was obtained from the Western Institutional Review Board (protocol #20152817). The ODxTT (US version) is a next-generation sequencing *in vitro* diagnostic test designed to detect somatic alterations in DNA and RNA with an amplicon based NGS methodology and utilizes tumor FFPE tissue samples.^12^ A comparison of the characteristics of the two assays, based on their publicly available specifications,^11^^,^^15^ are in Table 1.

**Table 1.** Characteristics of FoundationOne CDx, Oncomine Dx.

	FoundationOne CDx	Oncomine Dx (US version)
**Target enrichment approach**	Hybrid capture	Amplicon-based
**Total number of genes analyzed**	324 genes and select gene rearrangements from DNA	23 genes from DNA and fusions in *ROS1* from RNA
**Genomic signatures/biomarkers**	TMB MSI-H HRD signature	NA
**Specimen**	FFPE tissue	FFPE tissue
**DNA/RNA requirement**	50-1000 ng of DNA	10 ng of DNA and RNA

Abbreviations: CDx, companion diagnostic; DNA, deoxyribonucleic acid; FFPE, formalin-fixed paraffin embedded; LDT, laboratory developed test; MSI-H, microsatellite instability-high; NA, not applicable; ng, nanograms; RNA, ribonucleic acid; TMB, tumor mutational burden.

Source: FoundationOne CDx technical label^11^; Oncomine Dx technical label.^12^

### Primary analysis

This study retrospectively analyzed the identification of targetable biomarkers matched with FDA-approved or NCCN-recommended therapies using hybrid capture-based (F1CDx) and amplicon-based (ODxTT) next-generation sequencing (NGS). The prevalence of genomic alterations from a real-world cohort tested by F1CDx was compared to the prevalence of GA that would have been detected on ODxTT based on publicly available data on coverage specifications (Appendix B; https://www.accessdata.fda.gov/cdrh_docs/pdf16/P160045S019C.pdf).^15^ To simulate ODxTT detection, short variants were considered detectable only if both the gene and amino acid change matched ODxTT coverage specifications. For gene rearrangements, detections were considered only if fusion partner genes were listed on coverage specifications. This rule-based approach enabled a comparative analysis of real-world detection rates of actionable biomarkers by F1CDx vs the theoretical detection capacity of ODxTT.

## Results

### Frequency of targetable biomarkers in advanced NSCLC as detected by F1CDx

**Figure 1.**
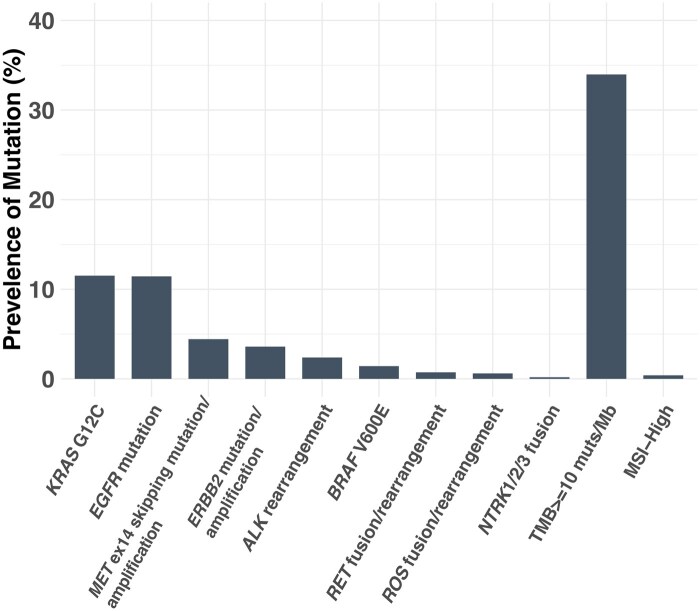
Prevalence of NSCLC samples with actionable biomarkers based on FDA approvals or NCCN guideline recommendations detected by F1CDx tumor profiling (*n* = 55 999).

We assessed the frequency of predictive biomarkers matched with FDA-approved or NCCN-recommended therapies in a large real-world genomic database of tissue samples from patients with NSCLC tested with F1CDx (*N* = 55 999). The genomic alterations examined are listed in Table 2. The analysis revealed that the most frequently identified alterations were in *KRAS* (12%, 6443/55 999)*,* followed by alterations in *EGFR* (11%, 6400/55 999), *MET* (4%, 2478/55 999), *ERBB2* (4%, 2012/55 999), *ALK* (2%,1330/55 999), *BRAF* (1%, 797/55 999), *RET* (0.7%, 410/55 999) *ROS1* (0.6%, 343/55 999), *NTRK1/2/3* (0.2%, 101/55 999), TMB>=10 mutations per megabase (34%, 19 021/55 999), and MSI-High (4%, 220/55 999; Figure 1). These counts reflect sample-level prevalence, meaning each number represents the samples with at least one detected alteration of a given biomarker.

**Table 2.** Biomarkers detected by FoundationOne CDx and should be detected on Oncomine Dx (based on ODxTT specification) for FDA-approved or NCCN-recommended therapies.

Biomarker	FoundationOne CDx	Oncomine DX (US version)	NCCN recommended/FDA approved therapies (as monotherapy or in combination with chemotherapy)
***EGFR* exon 19 deletion**	Detected	Would be partially detected	Afatinib, Amivantamab (+ Lazertinib), Dacomitinib, Erlotinib, Gefitinib, Osimertinib
***EGFR* exon 21 L858R mutation**	Detected	Would be detected	Afatinib, Amivantamab (+ Lazertinib), Dacomitinib, Erlotinib, Gefitinib, Osimertinib
***EGFR* G719X/S768I/L861Q mutation**	Detected	Would be partially detected	Afatinib, Dacomitinib, Erlotinib, Gefitinib, Osimertinib
***EGFR* exon 20 insertion**	Detected	Would not be detected	Amivantamab
***EGFR* T790M mutation**	Detected	Would not be detected	Osimertinib
***KRAS* G12C mutation**	Detected	Would be detected	Adagrasib, Sotorasib
***MET* exon 14 skipping alterations**	Detected	Would not be detected	Capmatinib, Crizotinib, Tepotinib
***MET* focal gene amplification**	Detected	Would not be detected	Capmatinib, Crizotinib, Tepotinib
***ERBB2* oncogenic mutation/amplification^a^**	Detected	Would be partially detected	Fam-trastuzumab deruxtecan, Ado-trastuzumab emtansine, Zongertinib
***ALK* rearrangement**	Detected	Would not be detected	Alectinib, Brigatinib, Ceritinib, Crizotinib, Ensartinib, Lorlatinib
***BRAF* V600E mutation**	Detected	Would be detected	Dabrafenib + Trametinib, Encorafenib + Binimetinib, Vemurafenib, Dabrafenib
***RET* fusion/rearrangement**	Detected	Would be partially detected	Pralsetinib, Selpercatinib, Cabozantinib
***ROS1* fusion/rearrangement**	Detected	Would be partially detected	Crizotinib, Entrectinib, Lorlatinib, Repotrectinib, Taletrectinib
***NTRK1/2/3* fusion**	Detected	Would not be detected	Entrectinib, Larotrectinib, Repotrectinib
**TMB >= 10**	Detected	Would not be detected	Pembrolizumab
**MSI high**	Detected	Would not be detected	Pembrolizumab

a *ERBB2* alterations refer to oncogenic or likely oncogenic at oncokb.org and include both mutation and amplification.

### Comparison of hybrid-based NGS and amplicon-based technologies in identifying targetable alterations

**Figure 2.**
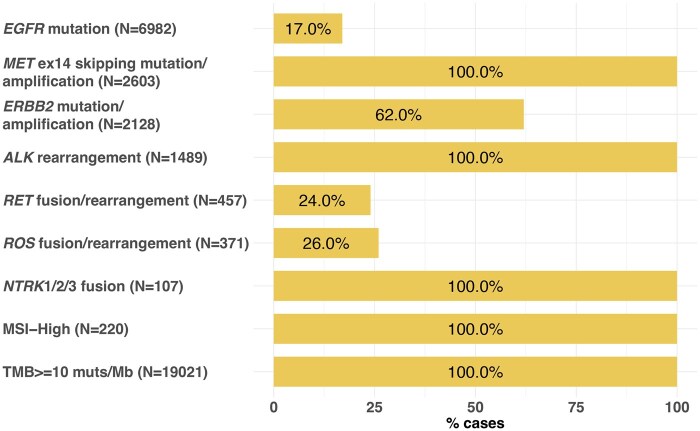
Bar graph of the actionable alterations in NSCLC (defined as those having matched FDA-approved or NCCN-recommended therapies) detected by F1CDx assay and not likely identified by the ODxTT assay based on publicly available data on coverage specifications.

We investigated the prevalence of targetable alterations identified by F1CDx and ODxTT (Table 1) at the alteration level. Using the rule-based approach described in the Methods section, we estimated the proportion of actionable alterations detected by F1CDx that would be theoretically identifiable by ODxTT. The analysis showed that, based on the specifications, ODxTT can identify all *BRAF* V600E and *KRAS* G12C with FDA-approved therapy detected by F1CDx, and the following gene alterations with FDA approved therapy at a lower proportion than F1CDx: *EGFR* (83% 5812/6982), *RET* (76% 347/457), *ROS1* (74% 275/371), and *ERBB2* (38% 810/2128). In addition, ODxTT had no coverage for actionable genomic findings in *ALK*, *MET*, or *NTRK1/2/3*, TMB–H, or MSI-High status (Figure 2). Overall, ODxTT would not have detected 64% (26 134/40 618) of actionable biomarkers detected on F1CDx.

At the sample level, ODxTT assay may fail to detect at least one of the clinically actionable biomarkers in this study in 42% (23 934/55 999) tissue samples based on what was reported by F1CDx (34% TMB-high [19 021/55 999]; 4% *MET* mutation [2478/55 999], 2% *ALK* rearrangement [1330/55 999]; 2% *ERBB2* mutation [1311/55 999]; 2% *EGFR* mutation [1151/55 999]; 0.4% MSI-High [220/55 999]; and <1% in *NTRK1/2/3* fusion [101/55 999], *RET* fusion/rearrangement [99/55 999], and *ROS1* fusion/rearrangement [92/55 999]). This means that if 100 samples were tested in the real world-setting with ODxTT alone, 42 samples may have at least one actionable result not available to the clinician when making treatment decisions for their patients, that would have been available by F1CDx.

## Discussion

The hybrid capture-based NGS test (F1CDx) and the amplicon-based NGS test (ODxTT) both offer improved detection of actionable biomarkers in advanced NSCLC compared with single gene testing.^10^^,^^16^^,^^17^ However, this study demonstrates that F1CDx captures more actionable markers compared with ODxTT based on the specifications of each test.^7^ Here, we observed that F1CDx is capable of detecting 64% of the actionable biomarkers that is not covered by the ODxTT. This includes the ability of F1CDx to detect genome-wide complex biomarkers such as TMB and MSI-H status, which may not be detected with smaller NGS panels such as the ODxTT. TMB has been shown to be able to not only identify patients who may more likely benefit from immunotherapy alone, but may also assist in the decision to add chemotherapy along with immunotherapy for selected patients with NSCLC.^18^ In sum, of 100 samples tested in the real world setting with ODxTT alone, 42 samples also tested with F1CDx may contain at least one actionable biomarker not covered with the ODxTT assay.

One of the main limitations of this study is that this was not a prospective test comparison. Rather, we compared the prevalence of genomic alterations from a real-world cohort tested by F1CDx with the publicly available coverage specifications on ODxTT. In addition, PD-L1 expression by immunohistochemistry is included in FDA labels and NCCN guidelines for advanced NSCLC but was not included in this analysis as PD-L1 IHC is not a component of either assay. Also, MET IHC has recently been approved as a CDx and is also not part of either assay. Of note, *NRG1* fusions has recently become an emerging rare therapeutic target; and so, is outside the scope of this analysis due to the testing time frame of this cohort. Future analysis should consider *NRG1* fusions with DNA and/or RNA CGP panels. Lastly, this study did not evaluate investigational markers that may only be actionable with novel therapies in clinical trials.

In conclusion, in advanced NSCLC, hybrid capture-based NGS, such as F1CDx, may capture more actionable genomic alterations as compared with ODxTT, an amplicon-based NGS. Future studies are needed to evaluate hybrid capture-based vs amplicon-based NGS in detecting actionable alterations across solid tumor types.

## Data Availability

The authors declare that all relevant aggregate data supporting the findings of this study are available within the article. In accordance with the Health Insurance Portability and Accountability Act, we do not have IRB approval or patient consent to share individualized patient genomic data, which contains potentially identifying or sensitive patient information and cannot be reported in a public data repository. Foundation Medicine is committed to collaborative data analysis and has well established and widely used mechanisms by which qualified researchers can query our core genomic database of >850 000 de-identified sequenced cancers. More information and mechanisms for data access can be obtained by contacting the corresponding author or the Foundation Medicine Data Governance Council at data.governance.council@foundationmedicine.com.
